# Prevalence of cancer and the benign call rate of afirma gene classifier in ^18^F‐Fluorodeoxyglucose positron emission tomography positive cytologically indeterminate thyroid nodules

**DOI:** 10.1002/cam4.3704

**Published:** 2021-01-15

**Authors:** Mayumi Endo, Jennifer A. Sipos, Matthew D. Ringel, Kyle Porter, Haikady N. Nagaraja, John E. Phay, Lawrence A. Shirley, Clarine Long, Chadwick L. Wright, Katie Roll, Fadi A. Nabhan

**Affiliations:** ^1^ Division of Metabolism, Endocrinology, and Nutrition University of Washington Seattle WA USA; ^2^ Division of Endocrinology, Diabetes, and Metabolism The Ohio State University Wexner Medical Center and Arthur G. James Cancer Center Columbus Ohio USA; ^3^ Center for Biostatistics Department of Biomedical Informatics The Ohio State University Columbus Ohio USA; ^4^ Division of Biostatistics College of Public Health The Ohio State University Columbus USA; ^5^ Department of Surgery The Ohio State University Comprehensive Cancer Center The Ohio State University Columbus Ohio USA; ^6^ Lexington Surgical Specialists Lexington KY USA; ^7^ The Ohio State University College of Medicine Columbus OH USA; ^8^ Wright Center of Innovation in Biomedical Imaging Division of Nuclear Medicine and Molecular Imaging Department of Radiology The Ohio State University Columbus Ohio USA

**Keywords:** ^18^Fluorine‐fluorodeoxyglucose, afirma, positron emission tomography, Thyroid Nodules

## Abstract

**Background:**

**^18^** F‐Fluorodeoxyglucose (FDG) positron emission tomography/computed tomography (PET/CT) positive (PET+) cytologically indeterminate thyroid nodules (ITNs) have variable cancer risk in the literature. The benign call rate (BCR) of Afirma Gene Classifier (Gene Expression Classifier, GEC, or Genome Sequence Classifier, GSC) in (PET +) ITNs is unknown.

**Methods:**

This is a retrospective study at our institution of all patients with (PET+) ITNs (Bethesda III/IV) from 1 January 2010 to 21 May 2019 who underwent Afirma testing and/or surgery or repeat FNA with benign cytology.

**Results:**

Forty‐five **(**PET+) ITNs were identified: 31 Afirma‐tested (GEC = 20, GSC = 11) and 14 either underwent surgery (*n* = 13) or repeat FNA (Benign cytology) (*n* = 1) without Afirma. The prevalence of cancer and noninvasive follicular thyroid neoplasm with papillary‐like nuclear features (NIFTP) including only resected nodules and ITN with repeat benign FNA (*n* = 33) was 36.4% (12/33). Excluding all Afirma “suspicious” non‐resected ITNs and assuming all Afirma “benign” ITNs were truly benign, that prevalence was 28.6% (12/42). The BCR with GSC was 64% compared to 25% with GEC (*p* = 0.056). Combining GSC/GEC‐tested ITNs, the BCR was higher in ITNs demonstrating low/very low‐risk sonographic pattern by the American Thyroid Association (ATA) classification and ITNs scoring <4 by the American College of Radiology Thyroid Imaging, Reporting and Data System (ACR‐TI‐RADS) than ITNs with higher sonographic pattern/score (*p* = 0.025).

**Conclusions:**

The prevalence of cancer/NIFTP in (PET+) ITNs was 28.6–36.4% depending on the method of calculation. The BCR of Afirma GSC was 64%. Combining Afirma GEC/GSC‐tested ITNs, BCR was higher in ITNs with a lower risk sonographic pattern.

## Graphical abstract

In this study, the prevalence of cancer and noninvasive follicular thyroid neoplasm with papillary‐like nuclear features in PET positive cytologically indeterminate thyroid nodules was 28.6–36.4% depending on the method of calculation. The benign call rate of Afirma GSC in these nodules was 64%. When combining Afirma GEC/GSC tested PET positive cytologically indeterminate thyroid nodules, the benign call rate was higher in nodules with a lower risk sonographic pattern.

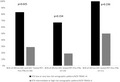

## 1 INTRODUCTION

Cytologically indeterminate thyroid nodules (ITNs), those classified as the Bethesda III/IV categories, pose a management challenge to physicians and patients. The risk of malignancy in these nodules is variable but is typically 15–30%.^1^ To avoid diagnostic surgery for what are ultimately benign ITNs, molecular diagnostic tests such as Afirma,^2^, ^3^ ThyroSeq,^4^, ^5^, ^6^ and ThyGeNEXT/ThyraMIR^7^ have been developed.

The risk of cancer in FDG‐positive nodules on PET/CT scans is estimated at 35%.^8^ However, the malignancy risk in FDG PET‐positive (PET‐pos) nodules that are ITNs by cytology is highly variable in the literature, ranging from 0 to 62%.^9^ The performance of molecular diagnostic tests in ITNs that are FDG‐positive on PET has not been reported. In this retrospective study, we sought to evaluate the prevalence of cancer in PET‐positive ITNs and the benign call rate of the Afirma test in these patients at a single institution.

## 2 METHODS

Clinical Data: This is a retrospective cohort study conducted at our institution that was approved by its Institutional Review Board. We queried the medical records from 1 January 2010 to 21 May 2019 and included in our cohort all patients who had FDG PET/CT studies that revealed a focal hypermetabolic thyroid nodule (regardless of SUV) within a year preceding an available fine needle aspiration biopsy of the same nodule classified as Bethesda III or IV on cytology. We further collected results of Afirma testing when performed, either by Afirma GEC (Dec 2011–July 2017) or GSC (July 2017–May 2019). The decision of whether to send samples for Afirma testing, versus surgery or observation was based on the clinical judgment of the treating physicians and patient preference. The FNA samples for cytology and molecular testing were obtained with a 23‐, 25‐, or 27‐gauge needle under ultrasound guidance. The molecular specimens were stored in a −60° celsius freezer. All samples were shipped at a temperature of −17° to −4° celsius to Veracyte, Inc. in South San Francisco, CA for testing. In those patients who underwent surgery, histopathology was examined by pathologists specializing in head and neck pathology. Noninvasive follicular thyroid neoplasm with papillary‐like nuclear features (NIFTP) was classified as “malignant” due to the current recommendations for management with hemithyroidectomy.^10^ Two endocrinologists retrospectively reviewed the ultrasound images of the nodules when available and classified them according to the ATA ultrasound stratification system^11^ and ACR‐TI‐RADS.^12^

Statistical Methods: Fisher's exact test was used to compare proportions and Wilson score method was used to compute 95% confidence intervals (CIs) for proportions. Mean ages were compared using a two‐sample *t*‐test; medians of size and volume of the nodules, and SUV max were compared using Wilcoxon test. Level of significance was set at 0.05.

## 3 RESULTS

### 3.1 Characteristics of nodules

Review of records revealed 52 FDG‐avid ITNs. Seven nodules were excluded because they did not undergo Afirma testing, repeat FNA (that yielded benign or malignant cytology), or surgery. The remaining 45 nodules (from 44 unique patients) were included in this analysis and consisted of those that underwent Afirma GSC (*n* = 11), Afirma GEC (*n* = 20), and those who either went to surgery without Afirma testing (*n* = 13) or had a repeat FNA that yielded benign or malignant cytology (*n* = 1) without Afirma testing. The mean patient age was 56 years and 44% were women. The median nodule size was 1.5 cm (IQR: 1.2–2.2), median nodule volume was 1.1 cm^3^ (IQR: 0.4–3.0). Fifty‐one percent of nodules (*n* = 23) were Bethesda III cytology and 49% were Bethesda IV cytology (*n* = 22). Forty‐two percent had Hürthle cell changes (among Bethesda III or IV nodules). For ultrasound classification, due to the small number of nodules, we considered ATA intermediate and high‐risk sonographic categories as one group (78%) and low and very low risk (18%) as another group. Similarly, the nodules were segregated by their ACR‐TI‐RADS score; those with a score of ≥4 (80%) were considered one group and those with a score of <4 (16%) as another group. Table 1 summarizes these characteristics and includes separately the characteristics of nodules that underwent Afirma GEC and GSC testing as well as nodules that underwent surgery or repeat FNA with benign or malignancy cytology without Afirma testing.

**TABLE 1:** Characteristics of nodules. 52 FDG PET‐positive cytologically indeterminate nodules were identified. Seven did not undergo Afirma testing nor underwent surgery or repeat FNA that yielded malignant or benign cytology and were excluded leaving 45 nodules for analysis: 20 underwent Afirma GEC testing, 11 underwent Afirma GSC testing, and 14 underwent surgery (*n* = 13) without Afirma testing or repeat FNA biopsy that was benign cytologically (*n* = 1)

Characteristic	All (*n* = 45)	GEC (*n* = 20)	GSC (*n* = 11)	No Afirma (*n* = 14)
Age, mean (SD) years	55.9 (12.9)	54.3 (12.9)	61.7 (11.1)	53.7 (13.6)
Gender, female	20 (44.4%)	11 (55.0%)	3 (27.3%)	6 (42.9%)
Nodule size (cm), median (IQR)	1.5 (1.2, 2.2)^a^	1.7 (1.2, 2.3)	1.5 (1.3, 2.3)	1.3 (1.1, 1.9)^a^
Nodule volume (cm^3^), median (IQR)	1.1 (0.4, 3.0)^a^	1.3 (0.6, 3.2)	0.9 (0.4, 4.2)	0.8 (0.3, 2.4)^a^
ATA sonographic risk				
Very low/low	8 (17.8%)	3 (15.0%)	3 (27.3%)	2 (14.3%)
Intermediate/high	35 (77.8%)	16 (80.0%)	8 (72.7%)	11 (78.6%)
Unknown	2 (4.4%)	1 (5.0%)	0 (0%)	1 (7.1%)
ACR‐TI‐RADS score				
<4	7 (15.6%)	3 (15.0%)	3 (27.3%)	1 (7.1%)
4–5	36 (80%)	16 (80.0%)	8 (72.7%)	12 (85.7%)
Unknown	2 (4.4%)	1 (5.0%)	0 (0%)	1 (7.1%)
Bethesda III nodules	23 (51.1%)	11 (55.0%)	6 (54.6%)	6 (42.9%)
AUS	14 (31.1%)	5 (25.0%)	4 (36.4%)	5 (35.7%)
FLUS	9 (20%)	6 (30.0%)	2 (18.2%)	1 (7.1%)
Bethesda IV nodules	22 (48.9%)	9 (45.0%)	5 (45.5%)	8 (57.1%)
HCN/SHN	13 (28.9%)	5 (25.0%)	1 (9.1%)	7 (50%)
SFN	9 (20%)	4 (20.0%)	4 (36.4%)	1 (7.1%)
Hürthle cell changes	19 (42.2%)	6 (30.0%)	5 (45.5%)	8 (57.1%)
SUVmax, median (IQR)	7 (5.3, 10.9)	6.3 (4.4, 7.9)	7.4 (6.2, 24.0)	7.2 (4.9, 11.9)

^a^ 1 missing.

### 3.2 Prevalence of thyroid cancer and NIFTP

We calculated the prevalence of malignancy first by including only patients with a histological diagnosis (*n* = 32) or a repeat FNA cytology that yielded either benign or malignant cytology (*n* = 1) (total number = 33) and this was 12/33 = 36.4% (95% CI: 22.2–53.4%). We then calculated the prevalence of malignancy assuming all Afirma benign ITNs were truly benign (*n* = 42) and the prevalence was 28.6% (95% CI 17.2–43.6%). There were no statistically significant associations between prevalence of cancer and age, gender, SUVmax, Bethesda III versus IV cytology, presence of Hürthle cell changes, sonographic appearance (by TI‐RADS and ATA), or size of nodules.

**FIGURE 1:**
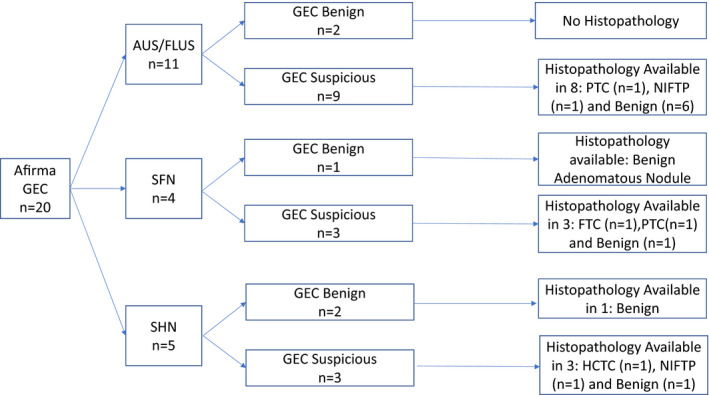
Summary of Afirma gene expression classifier (GEC) cytologically indeterminate thyroid nodules stratified by specific cytological diagnosis: (AUS: Atypical of undetermined significance, FLUS: Follicular lesion of undetermined significance, SFN: suspicious for follicular neoplasm, SHN: suspicious for Hürthle cell neoplasm) and GEC result (benign vs. suspicious) and histopathological diagnosis when available (PTC: papillary thyroid cancer, FTC: follicular thyroid cancer, NIFTP: Noninvasive Follicular Thyroid Neoplasm With Papillary‐Like Nuclear Features), HCTC: Hürthle cell thyroid cancer

**FIGURE 2:**
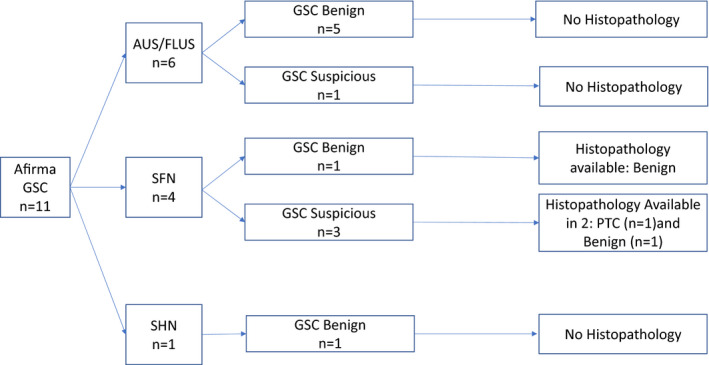
Summary of Afirma gene expression classifier (GSC) cytologically indeterminate thyroid nodules stratified by specific cytological diagnosis: (AUS: Atypical of undetermined significance, FLUS: Follicular lesion of undetermined significance, SFN: suspicious for follicular neoplasm, SHN: suspicious for Hürthle cell neoplasm) and GSC result (benign vs. suspicious) and histopathological diagnosis when available (PTC: papillary thyroid cancer, FTC: follicular thyroid cancer, NIFTP: Noninvasive Follicular Thyroid Neoplasm With Papillary‐Like Nuclear Features), HCTC: Hürthle cell thyroid cancer

The histology of malignant/NIFTP nodules included: four classic papillary thyroid cancer (PTC), one Hürthle cell variant PTC, three follicular variant PTC, one follicular thyroid cancer (FTC), one Hürthle cell thyroid cancer (HCTC), and two NIFTP. The prevalence of cancer in Afirma GEC suspicious nodules was higher (31.5%) than Afirma GSC suspicious nodules (11%) but this difference was not statistically significant (*p* = 0.371) and only two out of four suspicious Afirma GSC nodules went to surgery, one of which was malignant on histology (Figures 1 and 2). The prevalence of cancer in nodules that were surgically excised without Afirma testing was 38% (5/13). Indications for surgery in these 13 nodules (in 12 patients as two nodules were seen in one patient) included: patient preference in six, increase in size of nodule in one, diagnosis of thyroid cancer in one patient by a positive lymph node biopsy (yet the PET‐positive thyroid nodule itself was benign and patient had multifocal micro PTC elsewhere in thyroid), and the reason for surgery was not clearly stated in the remaining four patients.

### 3.3 Benign call rate, specificity, and PPV in PET‐pos ITNs that underwent Afirma testing

The benign call rate (BCR) in Afirma GEC versus Afirma GSC PET‐pos ITNs was 25% versus 64% (*p* = 0.056) (Table 2). Combining Afirma GEC and GSC, there were 12 benign Afirma nodules: seven with GSC and five with GEC. Three out of these 12 nodules (one GSC and two GEC) underwent surgery, all with benign pathology. The mean follow‐up of the remaining nine unoperated nodules was 16.8 months without changes in size or sonographic appearance.

**TABLE 2:** Cancer prevalence, benign call rate, positive predictive value (PPV), and specificity of Afirma‐tested, PET‐positive cytologically indeterminate nodules (ITNs)

Measure	Afirma GSC (*n* = 11)	Afirma GEC (*n* = 20)	*P*‐Value based on Fisher exact test
Cancer prevalence	11.1% (1/9)^a^	31.5% (6/19)^a^	0.371
BCR (Benign Call Rate)	63.6% (7/11)	25% (5/20)	0.056
PPV	50.0% (1/2)	42.8% (6/14)	1.00
Specificity	87.5% (7/8)	38.4% (5/13)	0.066

Sensitivity and NPV were 100% in both tests as we considered all benign Afirma ITNs truly benign and also all benign Afirma ITNs that were resected were negative for cancer.

^a^ Two nodules in Afirma GSC group and one in GEC group were not determined benign or malignant as they were suspicious on Afirma test and did not undergo surgery

**FIGURE 3:**
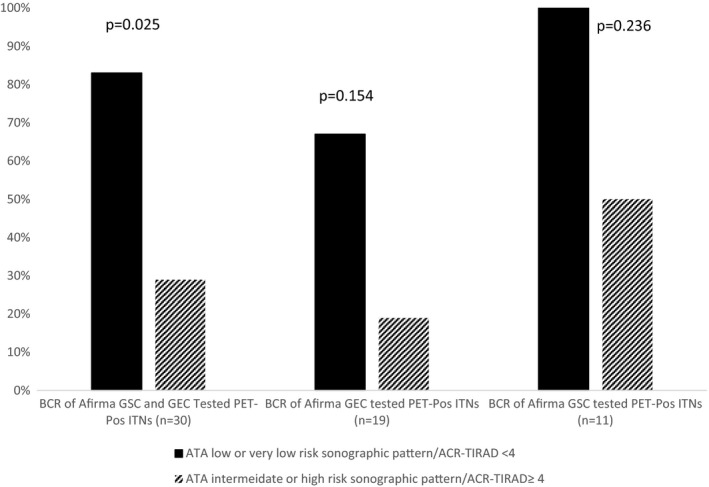
Benign call rate (BCR) of PET‐positive cytologically indeterminate nodules (ITNs) according to sonographic features. The number of ITNs with ATA low or very low sonographic pattern was the same as ACR‐TI‐RADS <4 score ITNs. The number of ITNs with ATA intermediate or high‐risk sonographic pattern was the same as ACR‐TI‐RADS ≥4 score ITNs

Sonographic features were available in 30 nodules (19 GEC and 11 GSC nodules). When combining both Afirma GEC and GSC‐tested ITNs, BCR was higher in ITNs with lower risk ATA ultrasound stratification (low or very low‐risk pattern) (83.3%) compared to intermediate or high‐risk ATA nodules (29%) and in ACR‐TI‐RADS <4 (83%) compared to ≥4 (29%) (*p* = 0.0256) (Figure 3). The same pattern was observed when evaluating Afirma GEC and GSC ITNs separately however, due to the small numbers of patients, the difference was not statistically significant (*p* = 0.154 and *p* = 0.236 for Afirma GEC and GSC‐tested ITNs, respectively) (Figure 3). There were no statistically significant associations between the BCR (in GEC or GSC or combined GEC and GSC) and age, gender, size of nodules, SUVmax, Bethesda diagnosis of III or IV, or Hürthle cell changes.

The specificity and PPV of the Afirma GEC compared to the Afirma GSC PET‐pos ITNs were 38% versus 88% (*p* = 0.066) and 43% versus 50% (NS), respectively (Table 2). NPV and sensitivity were 100% as we considered all Benign Afirma ITNs truly benign and all benign Afirma ITNs that went to surgery were truly benign. There were three patients with three suspicious Afirma nodules (one GEC and two GSC) that did not go to surgery: one due to patient reference (who had stable ultrasound at 14 months post FNA) and two due to progressive non thyroid‐related malignancy. (Both did not have additional follow‐up of thyroid nodule and both died at 3 months and 12 months after FNA biopsy due to progression of non‐thyroid‐related cancer.)

## 4 DISCUSSION

In the present study, we have studied the prevalence of cancer in PET‐pos ITNs and the benign call rate of Afirma GEC/GSC test. The risk of malignancy in a PET‐pos thyroid nodule is estimated at 35%.^8^ However, the risk of malignancy in PET‐pos nodules with an indeterminate cytology (Bethesda III/IV) is less clear and appears more variable. A systematic review showed that FDG PET positivity in an ITN carries a PPV of 0–62% with an overall cancer prevalence of 4–47%.^9^ In the present study, the prevalence of cancer in the cohort including only nodules that went to surgery was at 36.4%. When categorizing ITNs with a benign Afirma result as truly benign, the prevalence was 28.6%. The prevalence of cancer may have been influenced by the exclusion of 10 nodules (seven PET‐pos ITNs that did not undergo further testing by repeat FNA, surgery, or Afirma test and three suspicious Afirma nodules that did not undergo surgery) and it could have ranged between 23 and 42% if those excluded nodules were all benign or malignant, respectively.

Afirma GEC/GSC has been used in ITNs to determine whether conservative management with serial observation can be safely pursued. Given its high NPV and relatively low PPV, Afirma GEC was developed as a “rule‐out test”; however, the GSC, and the other available tests, have improved their positive predictive value with variability depending on the nodule selection and prevalence of cancer.^2^, ^6^, ^13^ The BCR is the percentage of molecular tests that results in a benign test result. For molecular tests with a high NPV such as Afirma and ThyroSeq,^6^, ^13^ the BCR typically reflects the percentage of patients that may be managed conservatively as if the cytology diagnosis is benign.

Improvement in BCR and PPV in GSC compared to GEC, when evaluating indeterminate thyroid nodules independent of PET positivity, has been previously reported.^14^, ^15^, ^16^ In the present study, which focuses on PET‐positive ITNs, there is also an improvement in the BCR and specificity in Afirma GSC compared to GEC (64% vs. 25% [*p* = 0.056] and 88% vs. 38% [*p* = 0.066], respectively). However, the PPV was similar for Afirma GSC (50%) compared to GEC (43%). This may be due to the higher prevalence of cancer in this group of nodules; however, the number of Afirma GSC suspicious nodules that went to surgery is small and there was a higher prevalence of cancer in the Afirma GEC / PET‐pos ITN (32%) compared to Afirma GSC/ PET‐pos ITN (11%).

The rate of malignancy in these Afirma‐tested ITNs may be influenced by the clinical practice pattern by different clinicians. As an example, those nodules with a reassuring U.S. pattern and indeterminate cytology may not undergo molecular testing due to the low likelihood of malignancy and instead may be offered serial sonographic monitoring. However, the patient population undergoing FDG PET imaging for another malignancy typically indicates that an incidentally identified metabolically active thyroid nodule is of lower priority than that seen in routine, non‐cancer patients. As such, the evaluation of PET‐positive thyroid nodules sometimes is geared toward finding ways to avoid delays in the treatment of their malignancy that initially necessitated the PET scan, including offering molecular testing when it ordinarily may not be performed. Consequently, the malignancy rate of these indeterminate nodules may be lower than those nodules identified by means other than FDG PET. Additional studies may be beneficial to examine the malignancy rate of this cohort.

We did not observe a statistically significant correlation between BCR and age, gender, nodule size, SUVmax, and sonographic features. However, when combining Afirma GEC and GSC nodules, there was a higher BCR in nodules with lower risk sonographic features. This was also seen in Afirma GEC and GSC groups when studied separately but was not statistically significant due to small numbers. The relatively low BCR in sonographically intermediate/high‐risk FDG‐positive ITNs suggests that this group of nodules may not benefit as much from molecular testing. The relationship between sonographic features and risk of cancer in FDG‐positive ITNs requires further investigation as higher risk sonographic features have been associated with higher risk of malignancy in indeterminate thyroid nodules^17^, ^18^ yet this has not been shown in all studies.^19^ It is also noted that there were 13 nodules in our cohort that went to surgery without Afirma testing and 38% (5/13) were malignant. It is not clear that the use of Afirma would have influenced those results.

There are several limitations of this study. The size of the cohort is relatively small and additional studies are required. Additionally, the Afirma GEC has now been replaced with the GSC. Since the GSC was implemented in 2017, the duration of follow‐up of ITNs interrogated with this test is shorter. Therefore, the possibility of falsely benign results cannot be excluded, although the impact is likely small given the high reported NPV of the test in other populations.^13^ Additionally, in this present study, 3/12 benign Afirma (GEC and GSC) underwent surgery with benign pathology and the average follow‐up on remaining nodules was 16.8 months, all nodules remained stable in size. Nevertheless, studies with longer follow‐up on these PET‐pos ITNs with benign Afirma are needed to help in determining the applicability of the Afirma GSC to safely avoid surgery in these patients without missing malignancy.

In conclusion, to the best of our knowledge, this is the first study that seeks to evaluate the benign call rate of Afirma testing in patients with cytologically indeterminate thyroid nodules that are also FDG PET‐positive. Additionally, compared to prior studies investigating the prevalence of cancer in such PET‐positive, cytologically indeterminate nodules, our population is one of the largest reported. We found that the prevalence of cancer ranged from 28.6% to 36.4% depending on which nodules were included in the calculation. We also found that the BCR of PET‐positive ITNs with the GSC was 64%, which indicates that surgical intervention may be avoided or delayed in these patients. However, the role of Afirma in FDG‐positive ITNs with intermediate or high‐risk ultrasound features requires further study due to a lower BCR in this group. Finally, in addition to larger studies with longer follow‐up to further confirm the true benign result of benign Afirma in this setting, future studies using other molecular diagnostic tests such as ThyroSeq V3 and ThyGeNEXT/ThyraMIR (6,7) are needed to evaluate further the performance of these tests in this context.

## CONFLICTS OF INTEREST

None.

## AUTHOR CONTRIBUTIONS

FAN, ME and JAS contributed to the conceptualization, methodology, design, data collection, data analysis, data interpretation writing and editing. MDR contributed to conceptualization, methodology, design, data analysis, data interpretation, writing and editing. KP and HNN contributed to methodology, data analysis, data interpretation, writing and editing. JEP and LAS contributed to data interpretation, writing and editing. CLW contributed to the design, writing and editing. CL and KR contributed to data collection. All authors approved the final version.

## Data Availability

All data generated or analyzed during this study are included in this published article or in the data repositories listed in References.
